# Food insecurity and child mental health in Masaka District, Uganda: Qualitative study using a realist thematic analysis

**DOI:** 10.1017/gmh.2026.10232

**Published:** 2026-05-22

**Authors:** Ibrahim Kasujja, Jacent Kamuntu Asiimwe, Hugo Melgar-Quinonez, Crick Lund, Tatiana Taylor Salisbury

**Affiliations:** 1 Centre for Global Mental Health, Health Services and Population Research Department, Institute of Psychiatry Psychology and Neurosciences, King’s College London, Denmark Hill, London, UK; 2 Department of Nutritional Science and Dietetics, Kyambogo University, Uganda; 3 Margaret A. Gilliam Faculty Scholar in Food Security, McGill Institute for Global Food Security, School of Human Nutrition, Faculty of Agricultural and Environmental Sciences, McGill University, Sainte Anne de Bellevue, Quebec, Canada; 4 Centre for Global Mental Health, Health Services and Population Research Department, Institute of Psychiatry Psychology and Neurosciences, King’s College London – Strand Campus, Denmark Hill, London, UK; 5 Alan J Flisher Centre for Public Mental Health, Department of Psychiatry and Mental Health, University of Cape Town, Rondebosch, Cape Town, South Africa

**Keywords:** Food insecurity, Child mental health, Internalising difficulties, Externalising difficulties, Mechanisms, Pathways, Hunger typologies, Explanatory model

## Abstract

Food insecurity and child mental health difficulties frequently intersect, yet the mechanisms and pathways connecting them remain underexplored in resource-limited settings. This study developed a contextually grounded explanatory model to examine these relationships within a single rural Ugandan setting. We conducted 12 focus group discussions with 36 teachers across four schools in Masaka district, drawing on their sustained observations of children’s food insecurity and mental health difficulties across a nine-month timeframe. Data were analysed using realist thematic analysis, supported by iterative coding, nine consensus meetings and member checking. Three interrelated pathways were identified. In the social causation pathway, food insecurity, manifested through hunger-related stress, food-related stigma and irregular or inadequate meals, preceded and contributed to mental health difficulties. In the social drift pathway, preexisting mental health difficulties among caregivers and/or children disrupted household functioning and food provision, increasing vulnerability to food insecurity. A bidirectional pathway captured recursive processes in which food insecurity and mental health difficulties co-evolved and reinforced one another over time. These pathways shaped children’s mental health and educational engagement within school environments. The findings offer context-specific insights that may inform research in similar settings. They also highlight the potential value of integrated, multi-level interventions, while underscoring the need for longitudinal and intervention research.

## Graphical abstract

**Figure:**
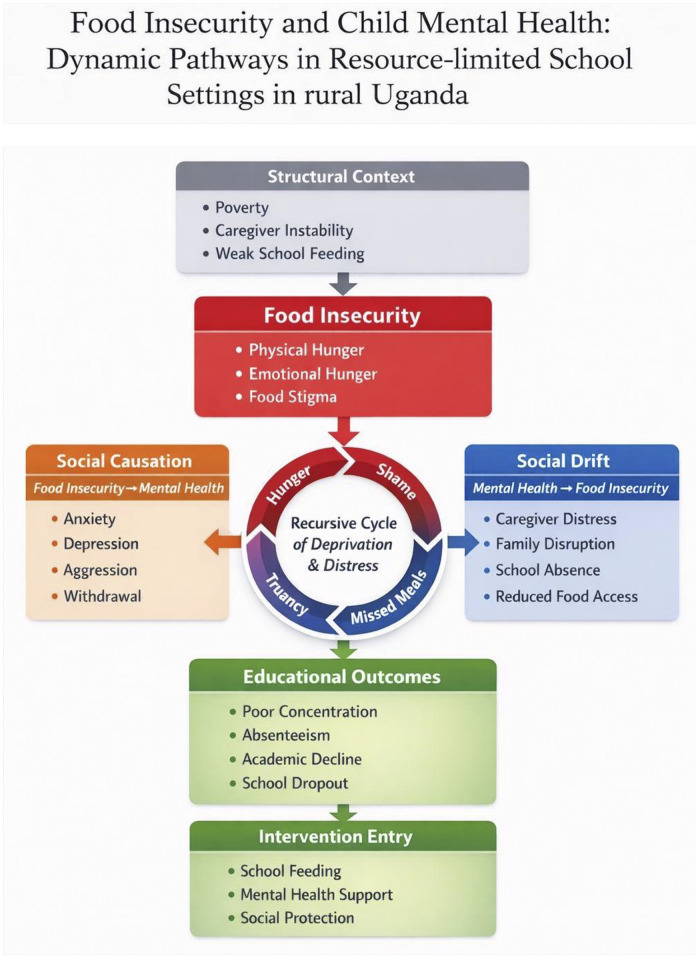

## Impact statement

This study advances understanding of how food insecurity and child mental health difficulties interact in resource-limited school settings by identifying three interconnected pathways of social causation, social drift and bidirectional feedback loops that shed light on how these dynamics unfold in everyday contexts. Food insecurity emerged as both nutritional and psychosocial conditions. Experiences of hunger, emotional distress and food-related stigma were linked to internalising difficulties (e.g., anxiety, withdrawal) and externalising behaviours (e.g., aggression, truancy), disrupting classroom engagement and academic performance. At the same time, mental health difficulties in children and caregivers undermined caregiving capacity, household stability and school participation, increasing vulnerability to food insecurity. These processes often co-occurred, forming reinforcing cycles in which hunger, shame, absenteeism and missed meals compounded disadvantage over time. By clarifying how these interactions unfold, the study shifts the focus from documenting associations to explaining the potential mechanisms that drive vulnerability in a low-resource context. The findings point to the importance of integrated, multi-level actions, spanning school, household and broader social protection systems, and highlight priorities for future research, including longitudinal and intervention studies to test these pathways.

## Introduction

Food insecurity is a major global health issue, affecting an estimated 2.3 billion people worldwide, with sub-Saharan Africa (SSA) experiencing the highest burden (Food and Agriculture Organisation (FAO), 2025). Uganda reflects this pattern. Structural poverty, climate shocks and limited social protection contribute to persistent household food insecurity, with significant implications for children’s daily functioning (Wiemers et al., 2024). At the same time, mental health difficulties often begin in early childhood and persist into adolescence, with a substantial burden of depressive and anxiety disorders among children (Opio et al., 2022).

Evidence from SSA consistently connects food insecurity with internalising and externalising mental health difficulties. A recent systematic review of 17 observational studies showed that food-insecure children are nearly three times more likely to experience mental health difficulties than their food-secure peers (Kasujja et al., 2025). Much of this evidence is derived from cross-sectional and quantitative studies, which are limited in their ability to explain the underlying mechanisms or processes through which these phenomena interact, particularly in everyday social contexts.

In addition, there is a relative scarcity of qualitative, contextually grounded research that captures how food insecurity and mental health difficulties are experienced, interpreted and navigated within children’s daily environments, especially in resource-limited school settings. As a result, key processes such as food-related stigma, peer dynamics, caregiving instability and school-level experiences remain insufficiently understood, despite their potential importance in shaping child mental health and well-being.

Children’s lived experiences vividly demonstrate this complex intersection. Food insecurity can manifest as arriving at school without meals, borrowing or stealing food, or experiencing stigma from peers or teachers, experiences that intensify anxiety, stress, shame and social withdrawal (Velardo et al., 2024). Conversely, parental or caregiver mental health difficulties may impair daily functioning, caregiving consistency, or household organisation, increasing vulnerability to food insecurity among children. These interconnected global issues of food insecurity and worse mental health emphasise the importance of shifting from merely documenting associations to comprehending underlying mechanisms.

Two theoretical perspectives shed light on these dynamic pathways. Social causation theory posits that food insecurity through hunger, food-related stigma, unpredictability and dietary monotony increases the risk of mental health difficulties (Ridley et al., 2020). Social drift theory suggests that pre-existing mental health difficulties in children or caregivers can undermine family functioning, household food provision and school participation, heightening vulnerability to food insecurity both at home and at school. While these perspectives are often studied separately, they may co-occur in practice, giving rise to dynamic, bidirectional processes that unfold over time (Lund et al., 2010; Patel et al., 2018).

Schools are critical settings for understanding these pathways, and represent a critical context in which these processes become visible. Despite the potential of schools to buffer socioeconomic adversity through school feeding programmes and psychosocial support, coverage of feeding programmes in resource-limited settings remains limited, reaching only 11% of school-age children in Uganda (World Food Programme, 2023). In resource-limited settings, many children attend school without reliable access to meals, or on an empty stomach, while also navigating social and emotional challenges linked to deprivation. This heightens psychosocial distress, impairs concentration and raises the risk of stigma (Kasujja et al., 2023).

Considering the significant prevalence of food insecurity and child mental health difficulties, and the limited understanding of the mechanisms connecting them in low-resource contexts, there is a need for research that moves beyond documenting associations to examining how and why these processes interact. Understanding the mechanisms connecting food insecurity and mental health difficulties is essential to inform integrated interventions that simultaneously address food insecurity, malnutrition, lack of psychosocial support and social protection in resource-limited contexts. Therefore, teachers, through their sustained interactions with children, are uniquely positioned to observe how food insecurity and mental health difficulties manifest and evolve in real-world settings.

This study, therefore, aimed to develop a contextually grounded explanatory model of the pathways connecting food insecurity and child mental health difficulties in resource-limited school settings in Uganda, drawing on teacher-reported child scenarios to identify and characterise social causation, social drift and bidirectional processes.

The objectives of this study are:

1. To explore how teachers perceive and describe the relationship between food insecurity and child mental health difficulties in resource-limited settings.
2. To identify and classify the psychosocial and behavioural manifestations of food insecurity among children, including internalising and externalising difficulties, and how this affects academic performance, classroom behaviour and school participation.
3. To develop a contextually grounded explanatory model that unpacks the mechanisms and/ or pathways connecting food insecurity and child mental health difficulties through the social causation, social drift and bidirectional pathways.

## Methods

### Study design

This study used an exploratory qualitative design to investigate teachers’ perceptions regarding the complex, bidirectional relationship between food insecurity and children’s mental health difficulties in a resource-limited context, namely Masaka District in Uganda. Focus group discussions (FGDs) were conducted to gather teachers’ perceptions, perspectives and insights into the daily experiences of children, particularly in last-mile school settings, which are defined as remote, resource-limited institutions that lack adequate infrastructure, staffing and educational support, serving children from low-income, food-insecure or conflict-affected households. Their geographical isolation from district centres and markets positions them at the “last mile” of educational service delivery (Hardman and Sandi, 2024), thereby placing children at a heightened risk of food insecurity and mental health difficulties.

Subsequently, a realist approach to thematic analysis (TA) was employed, enabling a detailed examination of teacher-reported child scenarios, emerging patterns and the core mechanisms and pathways connecting food insecurity to child mental health in both directions, thereby informing the development of a contextually grounded explanatory model that explains the social causation, social drift and bidirectional pathways.

### Study setting

This study was conducted in Masaka district, approximately 35 km from Masaka City Centre and 179 km from Kampala.

Masaka district has a high burden of child poverty. Nationally, 44% of children experience multidimensional poverty, rising to 57% in households with three or more children – a common demographic in Masaka. During 2019–2020, 23% of children in the district lived in monetary poverty, and child poverty exceeded 30% of the population (Uganda Bureau of Statistics (UBOS), 2024). These structural conditions are reflected in household food insecurity, where reliance on subsistence farming, limited dietary diversity and inadequate agricultural inputs remain prevalent (FAO, 2021; WFP, 2023).

Children under 18 make up approximately 58% of the district’s population, and many live in resource-limited households that struggle to meet their nutritional and educational needs. Local data reports that 13% of children under five in the district are severely stunted and 5% are severely underweight (United Nations Children Fund (UNICEF) and UBOS, 2023). Schools in Masaka span rural and peri-urban settings, making them both representative of broader national trends and a practical site for empirical research (Ministry of Education and Sports (MoES), 2021). Moreover, the region is part of Uganda’s central “cattle corridor,” where cycles of poverty, undernutrition and limited psychosocial support intersect, creating a convergence of risk factors ideal for exploring the linkages between food insecurity and child mental health (Akol et al., 2020).

### Participants and recruitment

Four primary schools were selected using stratified random sampling from a list of 203 government-registered schools in Masaka District, provided by the District Education Office. The list was first stratified by subcounty, and one school was randomly selected from each of four sub-counties to ensure geographic representation. Teachers from the selected schools were then asked for consent to participate in the study.

Of the 68 eligible teachers (aged 18–59 years) in the selected schools, four were primary seven teachers who were unable to participate in the qualitative study due to heavy workload, and an additional four teachers did not submit written consent within 72 h of receiving recruitment materials. These teachers were consequently excluded from the study. The response rate achieved was 88.2%.

Teachers who gave consent then completed a screening task, in which they indicated, on a 5-point scale, the number of meals the children they interacted with ate daily, ranging from 0 to 5 (i.e., early morning breakfast at home, school breakfast, school lunch, evening tea and supper). To facilitate further recruitment into FGDs, researchers balanced the sample by selecting an equal number of teachers who reported having had interactions with children consuming fewer than three meals per day and those who reported contact with children consuming more than three meals per day after screening. The number of FGDs were not predetermined but was guided by thematic saturation, that is, when no new insights emerged from teacher-reported child scenarios.

Twelve FGDs were conducted, each with three teachers, yielding a final sample of 36 teachers. Using maximum variation sampling, a broad range of views, perceptions and perspectives on the relationship between child food insecurity and child mental health difficulties were collected. Teacher participation continued until data saturation was achieved, identified after the twelfth FGD, with 95% data saturation based on the code frequency counts approach (Hennink and Kaiser, 2022).

### Data collection

Data were collected through teacher FGDs, each lasting approximately 90 min. The discussions were audio-recorded, and field notes were taken to capture non-verbal cues and group dynamics. Each FGD began with an overview of the study’s aims, followed by a review of the ground rules (confidentiality, respect and privacy) and an icebreaker. During the icebreaker, each teacher shared a brief, positive childhood memory related to food, such as a favourite family meal, a time when they felt particularly cared for, or an experience of sharing food with others. After the icebreaker, each teacher shared their perspective on the relationship between food insecurity and child mental health by describing detailed, real-life scenarios of children they had taught who experienced food insecurity and mental health challenges that had, retrospectively, affected their academic performance. The perceptions of teachers were explored through open-ended questions. FGDs were conducted in an unstructured manner, in Luganda, to allow teachers to express themselves freely and share culturally relevant experiences, probing more deeply into each teacher-reported child scenario to encourage natural conversation. Each teacher was allocated at least 20 min within each FGD to discuss a real-life scenario. The recordings were transcribed verbatim by five transcribers. The anonymised Luganda transcripts were then translated into English.

FGDs were selected to capture shared patterns and socially embedded processes in which food insecurity and mental health difficulties manifest within school environments. Teachers, through their sustained interactions with children, are uniquely positioned to observe recurring scenarios over time, making FGDs well-suited for eliciting comparative reflections, identifying common mechanisms and generating collectively validated interpretations. This approach aligns with the study’s aim of developing a contextually grounded explanatory model, as group interaction enabled participants to build on one another’s accounts and surface cross-cutting patterns that may not emerge through individual interviews alone. However, while FGDs provide insight into shared experiences and school-level dynamics, they may be less suited to capturing deeply individualised narratives or detailed household-level processes.

### Analytical approach

**Figure 1.**
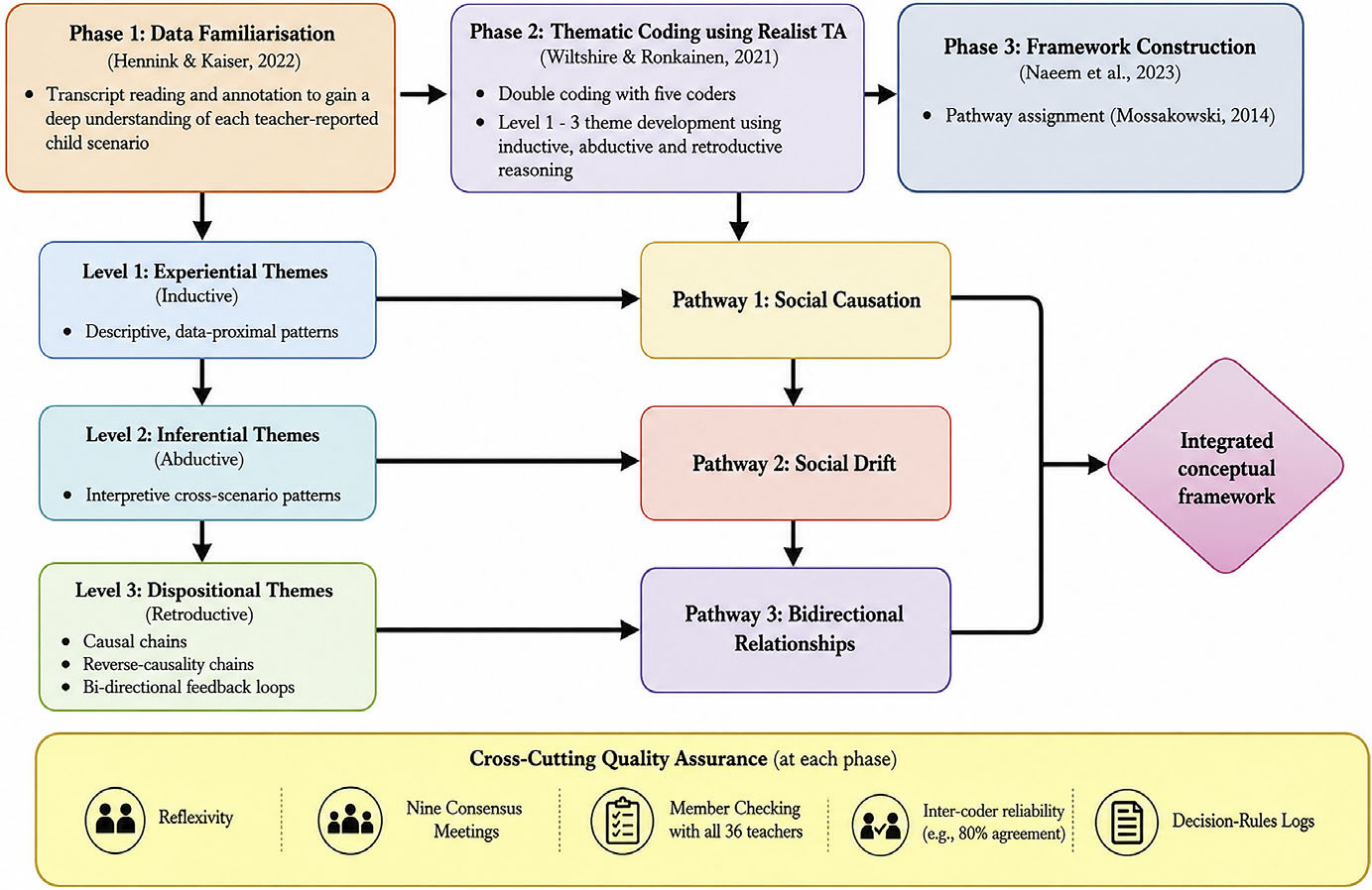
Flowchart illustrating the development of a contextually grounded explanatory model connecting social causation, social drift and bidirectional pathways. Figure 1. long description.

To ensure rigour and consistency, five coders conducted rotating double-coding: each transcript was independently coded by two coders rather than the entire team. Coder pairings were rotated across transcripts to balance workload, minimise bias and capture diverse interpretive perspectives. Following the double-coding process, the team held nine consensus meetings to discuss discrepancies, refine code definitions and finalise the coding framework. Decision rules were documented in shared Excel worksheets on the OneDrive platform, and codes were finalised once 80% inter-coder agreement was achieved (Maxwell, 2010). Reflexivity was maintained through ongoing discussions of positionality and interpretive assumptions, while member checking with all 36 teacher participants further enhanced interpretive validity, as feedback on the English-translated scenario summaries informed refinements to themes across the three analytical levels (Birt et al., 2016). These quality-assurance procedures were cross-cutting, spanning phase 1 (data familiarisation), phase 2 (thematic coding using realist TA) and phase 3 (explanatory model construction), to maintain coherence and transparency throughout the analytical process (see Figure 1).

#### Phase 1: Data familiarisation

In the first phase, coders engaged in *data familiarisation* (Hennink and Kaiser, 2022), carefully reading and annotating all transcripts to develop a holistic understanding of each teacher-reported child scenario, thereby establishing a strong foundation for theme extraction in the subsequent phases of realist TA.

#### Phase 2: Thematic coding

In the second phase, a realist TA framework, as described by Wiltshire and Ronkainen (2021), was used to identify, interpret and theorise mechanisms connecting food insecurity and child mental health difficulties. This phase unfolded through three complementary modes of reasoning – inductive, abductive and retroductive – each corresponding to progressively higher levels of analytical abstraction.

*Level 1. Experiential themes: Inductive reasoning* was used to identify experiential themes, teachers’ descriptive accounts of children’s lived experiences, intentions, hopes, concerns, beliefs and feelings, directly from the data, without reference to pre-existing categories or theoretical models (Braun and Clarke, 2006). These themes provided descriptive summaries of observable food insecurity experiences and child mental health difficulties as narrated by teachers, remaining close to the data. For instance, scenarios in which children begged for food or were mocked for not bringing lunch were coded as experiential themes. These themes constituted the empirical foundation of the TA and were subjected to member checking (respondent validation) to ensure interpretive validity. This data-driven approach uncovered experiential patterns as they naturally appeared across teacher-reported child scenarios.

*Level 2. Inferential themes: Abductive reasoning* was used to develop inferential themes, moving beyond description toward interpretation. Abduction involves forming interpretations that most plausibly explain observed patterns, especially those that are unexpected or complex, by proposing the best explanatory fit (Peirce, 1931–1958; Timmermans and Tavory, 2012). These inferential themes captured analytical patterns across multiple teacher-reported scenarios, explaining recurring psychosocial and behavioural dynamics such as school disengagement linked to hunger and stigma, or aggression as a response to food denial after begging. Through this process, the research team synthesised cross-cutting observations into coherent explanatory insights, which were again subjected to member checking to confirm interpretive accuracy.

*Level 3. Dispositional themes: Retroductive reasoning* was used to derive dispositional themes—theoretical inferences about the underlying mechanisms and contextual properties that generate both food insecurity and child mental health phenomena. Retroduction involves reasoning backwards from observed outcomes to identify the causal structures that must exist for these outcomes to occur (Danermark et al., 2002; Bhaskar, 2014). Three hunger typologies emerged as key dispositional themes: physical hunger, defined as the physiological drive to eat in response to energy deficits such as an empty stomach or low blood sugar (Lowe and Butryn, 2007; Stevenson et al., 2023). Emotional hunger, defined as the urge to eat triggered by negative emotional states, such as stress, sadness, or anxiety, rather than a physiological need (Macht, 2008), and hedonic hunger, defined as the desire to consume palatable foods for pleasure in the absence of energy need, connected to reward pathways (Lowe and Butryn, 2007). These typologies offered greater explanatory depth to the analysis by showing how hunger goes beyond physiological deprivation, involving emotional and psychological processes that influence children’s emotional and behavioural functioning, as well as their mental health outcomes. *Dispositional themes* thus represented explanatory propositions about latent causal mechanisms and contextual conditions underlying both food insecurity and mental health difficulties. These included causal chains, reverse-causality chains, bidirectional feedback loops and mutually reinforcing causal circuits, generated by temporal sequencing of events in each teacher-reported scenario and identifying initiating conditions. The initiating conditions and temporal sequence of the teacher-reported child scenarios quoted across different pathways are provided in Supplementary Table S1.

Through retroductive reasoning, the research team hypothesised the underlying processes, herein referred to as mechanisms, connecting these dynamics, and identified latent structures such as household poverty, caregiver absence and institutional gaps in school meal provision that plausibly shaped both children’s lived experiences of food insecurity and mental health difficulties, as well as teachers’ interpretations of events within each social causation, social drift and bidirectional loop scenarios.

This layered, realist approach enabled the coders to progressively deepen their analysis, from what was said and observed to why these patterns might exist, thereby providing greater insight into how food insecurity and mental health difficulties are interconnected within resource-limited, last-mile school settings. To enhance conceptual clarity, we distinguish between pathways, mechanisms and causal chains. Pathways capture the directional relationships between food insecurity and mental health difficulties (e.g., social causation, social drift and bidirectional pathways). Mechanisms refer to the processes, such as hunger, stigma, caregiving disruption and behavioural reactions, through which these relationships are enacted. Causal chains are constructed through temporal ordering, representing the stepwise sequences through which these mechanisms unfold over time. Pathways are therefore differentiated not only by direction but also by differences in initiating conditions and temporal sequencing, rather than by mechanisms alone.

#### Phase 3: Explanatory model construction

In the third phase, coders categorised each teacher-reported child scenario into one of three main pathways and clustered *experiential*, *inferential* and *dispositional* themes. This categorisation drew on established social causation and social drift principles and was applied using explicit decision rules to ensure consistency and conceptual clarity in theory-driven coding (Naeem et al., 2023). To ensure conceptual clarity, pathways were distinguished by the temporal ordering that emerged from each teacher-reported child scenario and its initiating conditions.

*Pathway 1. Social causation:* This pathway was assigned when 80% intercoder agreement was reached between coders that food insecurity was the initiating factor leading to the child’s mental health difficulties in each teacher-report child scenario. For example, irregular meals, the absence of a packed lunch, eating only one meal a day and shame or ridicule related to inadequate access to safe, affordable and nutritious food would have preceded and plausibly precipitated psychological distress (e.g., anxiety, withdrawal) or behavioural problems (e.g., aggression, inattention). Therefore, the social causation pathway was identified, where food insecurity preceded and contributed to the emergence of mental health difficulties.

*Pathway 2. Social drift*: This pathway was assigned when 80% intercoder agreement was reached between coders that preexisting mental health difficulties—either in the child or a primary caregiver—appeared to increase food insecurity (as a stand-alone), or if they appeared to diminish family or household functioning, creating family instability or school disengagement, thereby increasing vulnerability to food insecurity. For instance, caregiver mental illness leading to neglect, or child mental distress resulting in school absenteeism (and thus missing meals), would exemplify this downward drift into nutritional deprivation (e.g., starvation) and food insecurity. Therefore, the social drift pathway was identified, where pre-existing mental health difficulties preceded and contributed to worsening food insecurity.

*Pathway 3. Bidirectional loop*: This pathway was assigned when 80% intercoder agreement was reached between coders that the teacher-reported child scenarios describe negative, sometimes cyclical, feedback loops, in which food insecurity and mental health difficulties co-evolved and mutually exacerbated each other. For example, physical hunger would have triggered peer stigma, which in turn led to truancy or disengagement, resulting in missed school meals and worsening psychological hunger, further amplifying psychological symptoms such as shame, anxiety, or social isolation. Therefore, the bidirectional pathway was identified where food insecurity and mental health difficulties co-evolved over time in recursive feedback loops, without a clear single initiating condition.

This systematic application of causal typologies enabled this qualitative study to distinguish between distinct but interrelated pathways connecting food insecurity and child mental health difficulties in resource-limited contexts. It also offered a robust analytical framework for the subsequent development of a contextually grounded explanatory model.

## Results

### Participant profile

A total of 36 teachers (mean age of 40.7 years; 21 females [58.3%]) participated in 12 FGDs, each comprising three teachers. Each teacher provided at least one child scenario related to food insecurity and mental health difficulties. Twenty-six teacher-reported child scenarios involved boys (72.2%) and focused on children with a mean age of 10.8 years (age range: 7 to 13 years old), often considered to be in middle childhood.

Teachers reported that seventeen children were from nuclear families (47.2%), eleven were from extended families (30.6%), with four children from both child-headed households and homeless conditions (each 5.6%). Thirty-five teacher-report child scenarios (97.2%) featured neglected children or children involved in child labour.

### Household context and caregiving dynamics

Teachers-reported child scenarios revealed that 20 (55.6%) children had grandparents as primary caregivers and 10 (27.8%) had step-parents as primary caregivers, indicating that most children came from extended families. Throughout various teacher-reported child scenarios, teachers reported that caregiving arrangements were frequently characterised by instability, often disrupted by factors such as illness, substance abuse, alcoholism, parental separation, caregiver upheaval or extended absences. Additionally, teachers emphasised that unstable caregiving arrangements exacerbated food insecurity, intensified child mental health difficulties and hindered daily functioning in children.

This study examined pathways considering household context and caregiving dynamics, which we present as analytical distinctions rather than mutually exclusive categories, reflecting the complexity of children’s lived experiences in resource-limited contexts.

### Social causation: Food insecurity preceding child mental health difficulties

Nineteen teachers (52.8%) reported cases in which household and/or school-based food insecurity was the initial condition preceding child mental health difficulties, based on the temporal ordering of events within teacher-reported social causation scenarios (see Table 1).

**Table 1.** Influence of food insecurity on child mental health: inferential and dispositional themes Table 1. long description.

Inferential themecategory 1.[Scenario] Food insecurity indicators (Severity)	Inferential themecategory 2. Internalising difficulties	Inferential theme category 3. Externalising difficulties	Dispositional themecategory 1.Other contextual variables	Dispositional themecategory 2.Causal chains [Pathway]
[1] Goes hungry to bed, comes to school hungry, steals food, no food from home (severe food insecurity^‡^)	Anxiety*, hopelessness, loss of confidence, social isolation*, social withdrawal	Arguments, fighting, food theft**, vulgar language, and disruptive behaviour	[Parental illness, household instability]^a^ [Social exclusion, peer avoidance]^b^, (Teacher punishment)^c^	Physical hunger → anxiety, irritability, arguments, food theft, social withdrawal, academic decline [Social causation]^d^
[2] Frequently left without enough food, skips meals, physical weakness, collapses from hunger (severe food insecurity^‡^)	Sadness, mutism, suicidal thoughts*, and depressed mood*.	Defiance, social marginalisation, truancy**	[Parental neglect, family disruption]^a^ [Social exclusion, peer mockery, bullying, absenteeism]^b^,[Peer attitudes, Teacher response]^c^	Physical hunger → peer marginalisation, absenteeism → mutism, suicidal thoughts[Social causation]^e^
[3] Only eats supper, arrives at school hungry, brings only boiled cassava, begs and steals food (severe food insecurity^‡^)	Anxiety*, worthlessness, low self-esteem*, sense of misfortune	Begging, food theft**, peer conflict	[Homelessness, family deprivation]^a^ [Bullying victimisation, peer conflict]^b^, [Peer mockery vs. support]^c^	Emotional hunger → excessive worry, anxiety, worthlessness, begging, stealing food, social withdrawal, academic decline, dropout [Social causation]^d^
[4] Only one meal per day, stays in the classroom during breaktime, hopes the teacher will give food (severe food insecurity^‡^)	Social/emotional withdrawal*, anxiety*, low mood, forgetfulness, drowsiness	Attention-seeking, begging others for food, aggression**	[Parental separation, caregiver neglect]^a^ [Teacher attention (potential), isolation]^b^, [Teacher attention/neglect]^c^	Physical hunger → anxiety, low mood, emotional withdrawal, attention-seeking, drowsiness, reduced self-esteem, academic decline [Social causation]^d^
[5] Brings only sweet potatoes from home, mocked for meagre food, goes to bed hungry, avoids eating at home (severe food insecurity^‡^)	Depressed mood, low self-esteem*, frustration, social isolation and withdrawal*, lost motivation	Begging**, food theft**, anger, fighting	[Household instability, parent/caregiver alcoholism]^a^ [Bullying, mockery, isolation]^b^, [Peer exclusion]^c^	Emotional hunger → begging, stealing, anger, bullying, self-esteem decline, loss of motivation/talent [Social causation]^d^
[6] No food to bring to school, food only provided for supper, steals food, chronic hunger (severe food insecurity^‡^)	Withdrawal, social isolation*, academic/language decline	Food theft, aggression**, truancy	[Parental loss, caregiver neglect]^a^ [Social exclusion, loss of friendships]^b^, [Teacher/peer support]^c^	Physical hunger → aggression, anti-social behaviour, absenteeism, academic decline, isolation, repetition [Social causation]^d^
[7] Only supper at home, visible emaciation, begs for food at school, and comes to school weak (severe food insecurity^‡^)	Worry, fear, depression, self-blame, loneliness, sadness*, withdrawal	Begging for food**	[Family exploitation, domestic violence, household instability, learning poverty]^a^ [Peer mockery, social rejection]^b^, [Teacher/peer support]^c^	Physical hunger → worry, depression, self-blame, social withdrawal, academic decline, repetition [Social causation]^d^
[8] Only yams and hot water (monotonous diet), often work for food, frequently faint after skipping meals (severe food insecurity^‡^)	Worry, sadness*, distress, discouragement*, loss of confidence	Truancy**	[Extreme poverty, frail caregiver]^a^ [Child labour, school absences]^b^, [Teacher or community aid]^c^	Emotional hunger → sadness, worry, maladaptive behaviour, school absences, lost talent [Social causation]^d^
[9] No food from home, begs and steals food, works to obtain food for survival (severe food insecurity^‡^)	Low self-esteem, stress, depressed mood*, self-harm*	Begging, food theft**, and social conflict	[Parental separation, household instability, substance abuse]^a^ [Child labour, social exclusion]^b^, [School discipline, community support]^c^	Physical hunger → neglect, child labour, exclusion → depression, disruptive behaviour, self-harm [Social causation]^e^
[10] Only one type of food (cassava), steals from classmates, lingers near the staffroom for leftovers (severe food insecurity^‡^)	Sadness, mutism*, social isolation	Begging, stealing**	[Parental separation, neglect, and alcoholism]^a^, [Peer mockery, rejection, abuse]^b^, [Peer attitudes]^c^	Emotional hunger → sadness, begs, steals, mutism, masking behaviour, loss of friendships, social isolation [Social causation]^d^
[11] Skips breakfast/lunch, steals and fights for food, fetches water for neighbours to get food (severe food insecurity^‡^)	Worry, anxiety, sadness, self-harm*	Food theft, aggression**, fighting, truancy	[Orphanhood, deprivation, child labour]^a^ [Peer conflict, absenteeism]^b^, [Teacher/school discipline climate]^c^	Physical hunger → fighting, absenteeism → self-harm, school dropout [Social causation]^e^
[12] Eats pineapples for meals, works for food, hides food due to shame, suffers persistent headaches from diet (severe food insecurity^‡^)	Hopelessness, depressed mood, emotional distress*, shame, social isolation, forgetfulness, crying.	Hiding food, lateness, truancy**, incomplete work, disengagement	[Orphanhood, caregiver neglect]^a^ [Lateness, absenteeism, low motivation]^b^, [Family/teacher understanding]^c^	Physical hunger → lateness, absenteeism → hopelessness, school dropout [Social causation]^e^
[13] Often hungry, cannot eat school porridge without sugar, steals, and engages in risky behaviours for food (severe food insecurity^‡^)	Blunt affect, shyness, shame, social isolation*, disinterest, inattentiveness	Stealing, irritability, risky sexual behaviour**	[Family instability]^a^ [Peer influence, risky behaviour]^b^, [Peer group norms, school climate]^c^	Emotional hunger → peer influence, risky sexual behaviour → school dropout [Social causation]^e^
[14] Never brings food to school, eats only supper at home, begs and steals others’ food (severe food insecurity^‡^)	Anxiety, fear*, depressed mood	Food theft**	[Parental separation]^a^ [Bullying, peer rejection, punishment]^b^, [Teacher support]^c^	Physical hunger → bullying, rejection, isolation → anxiety, academic decline [Social causation]^e^
[15] Eats only boiled cassava for every meal, always finishes quickly because it is not enough, ostracised for stealing food (severe food insecurity^‡^)	Anxiety, social withdrawal*, low self-esteem, self-guilt, social isolation	Food theft**, binge eating, and craving	[Poverty, malnutrition]^a^ [Peer exclusion, stigma, malnutrition]^b^, [Stigma/exclusion vs. sharing]^c^	Emotional hunger → anxiety, food theft, social withdrawal, academic decline [Social causation]^d^
[16] Does not often eat supper, begs for food at break and lunch time (severe food insecurity^‡^)	Fatigue, worry, distress, low self-esteem, social withdrawal*	Begging for food**	[Family conflict, neglect, alcoholism]^a^ [Social exclusion, incomplete work, morale]^b^, [Peer/teacher support, school climate]^c^	Physical hunger → begging, exclusion → distress, academic difficulty [Social causation]^e^
[17] Eats only cassava or sweet potatoes, steals food, fixates on the school kitchen for a chance at food (moderate food insecurity^‡^)	Anxiety, panic, guilt, low self-esteem, shame*, social withdrawal, isolation	Food theft, aggression, peer conflicts**	[Poverty, family instability]^a^ [Bullying, peer rejection, non-attendance]^b^, [Peer attitudes, Teacher response]^c^	Physical + hedonic hunger → obsession with food, steals food, maladaptive behaviour, withdrawal, shame, academic decline, school withdrawal [Social causation]^d^
[18] Brings unsweetened cassava porridge to school, begs food from neighbours, avoids begging peers out of shame, visible bones due to wasting (severe food insecurity^‡^)	Anxiety, low self-esteem, self-loathing*, hopelessness, loneliness	Disinterest in play, truancy**	[Poverty, malnutrition]^a^ [Social exclusion, loneliness]^b^, [Peer attitudes, teacher response]^c^	Physical hunger → worry, anxiety, self-loathing, absenteeism, drowsiness → social exclusion, loneliness, academic decline [Social causation]^d^
[19] Eats only bean leaves or raw cassava, scavenges for leftovers (severe food insecurity^‡^)	Fatigue, hopelessness, self-harm, isolation	Stealing, aggression**, fighting	[Parental neglect]^a^ [Peer mockery, bullying, child labour]^b^ [Community/family, peer attitudes]^c^	Emotional hunger → bullying, child labour → self-harm, dropout[Social causation]^e^

→ (red arrow) indicates a direct causality.

→ (blue arrow) indicates an indirect causality.

‡ An inferential theme extracted through abductive reasoning.

* A most dominant internalising difficulty.

** A most dominant externalising difficulty.

a Contextual themes not in a causal sequence.

b Contextual themes lying in a causal sequence.

c Contextual themes strengthening or weakening the causal interaction.

d Direct pathway.

e Indirect pathway.

#### Direct social causation

Seven (19.4%) reported frequent meal skipping and visible signs of malnutrition (e.g., wasting, stunting, pale appearance, swollen belly) and four teacher-reported scenarios (11.1%) described persistent hunger and a lack of food at school.

Teachers linked frequent meal skipping and malnutrition to internalising difficulties (e.g., anxiety, depression, social withdrawal, low self-esteem, hopelessness, self-harm and suicidal ideation) and externalising difficulties (e.g., aggression, stealing, disruptive behaviour, truancy, risky sexual behaviour and other antisocial acts) among children in resource-limited settings.

Teachers reported that some children internalised their hunger through social withdrawal and declining confidence, while others exhibited more overt psychological distress and risk behaviours.

An 11-year-old boy living with his single mother arrived at school hungry every day. Severe food insecurity at home, combined with his mother’s illness, eroded his mental health: he became anxious and irritable, argued with classmates, and sometimes stole food. Punishment by teachers and avoidance by peers deepened his isolation, reduced confidence, increased absences, and precipitated academic decline, culminating in school withdrawal.^**1**^ (Female teacher, 32 years).

Although this boy’s experience demonstrated how physical hunger could cause anxiety, isolation and withdrawal from school, another boy’s experience highlighted even more serious mental health consequences, including self-harm.

An 11-year-old orphaned boy living with grandparents in a severely food-insecure household frequently skipped breakfast and lunch, stole food at school, and fought when reported. When he could not find food, he grew anxious and once attempted to harm himself by drinking paraffin. Attendance fell to two or three days per week as he fetched water for neighbours in exchange for food. His performance declined, and he repeated the class; eventually, he dropped out.^**2**^ (Male teacher, 45 years).

The direct social causation pathway operated through making food insecurity an immediate and proximal driver of children’s mental health and behavioural difficulties, with the effects of food insecurity on mental health appearing to occur without the need for intermediary social or behavioural processes.

#### Indirect/mediated social causation

Eight teachers (22.2%) reported that food insecurity impacted child mental health through various mediators: bullying, exclusion, absenteeism, peer rejection, child labour, risky behaviours and academic decline. Teachers reported that food-related stigma from eating non-preferred foods and physical hunger caused anxiety, irritability or aggression in children, leading to social exclusion and teacher punishment. Furthermore, teachers reported that these factors together contributed to absenteeism, poor academic performance and high school dropout rates.

A 13-year-old girl living in a severely food-insecure household illustrates a mediated social causation pathway, where structural deprivation operated through psychosocial and behavioural mediators. Persistent hunger and dietary monotony generated hunger-related stress, prompting survival strategies such as stealing money, associating with risky peers, and engaging in exploitative relationships to access food. These adaptive responses increased her exposure to risk, culminating in early pregnancy. Subsequently, shame, stigma, and social isolation further mediated disengagement from school, while ongoing hunger-related stress impaired concentration and learning. Together, these interconnected pathways led to declining academic performance and eventual school dropout.^**3**^ (Female teacher, 41 years)

While this girl’s experience highlighted how food insecurity indirectly shaped mental health through survival strategies and social risks, another boy’s experience demonstrated the same pathway operating through anxiety and peer rejection.

A 10-year-old boy from a child-headed household arrived hungry, having no packed food. Chronic hunger heightened anxiety and fear, prompting food theft that triggered bullying, social isolation, and teacher punishment. Peer rejection and disciplinary responses further undermined focus and well-being, with hunger underlying all difficulties.^**4**^ (Female teacher, 46 years).

In some cases, broader structural factors such as climate variability contributed to household food insecurity, which was then experienced by children through hunger, emotional distress and school disengagement.

A 12-year-old boy from a farming household experienced food shortages following poor harvests linked to irregular rainfall. Reduced food availability at home led to hunger, anxiety, and withdrawal in school. He avoided participating in class, was frequently absent during peak labour periods, and his academic performance declined.^**5**^ (Male teacher, 34 years).

The mediated social causation pathway operated through behavioural adaptations (e.g., survival strategies), social responses (e.g., stigma, punishment) and broader structural factors such as climate-related disruptions to household food production. These processes interacted to shape children’s mental health and educational outcomes, with environmental shocks affecting food availability at the household level and subsequently experienced by children through hunger, emotional distress and school disengagement.

### Social drift: Parental and child mental health difficulties preceding increased food insecurity

Five teachers (13.9%) reported cases where parental and/or caregiver mental illness, and in some instances, child mental health problems, were the initial conditions preceding household food insecurity and/or school-based food insecurity, based on the temporal order of events within the teacher-reported social drift scenarios (see Table 2).

**Table 2.** Influence of mental health difficulties on food insecurity: inferential and dispositional themes Table 2. long description.

Inferential theme category 1. [Scenario] Internalising difficulties	Inferential theme category 2. Externalising difficulties	Inferential theme category 3. Food insecurity indicators [Severity]	Dispositional theme category 1. Other contextual factors	Dispositional theme category 2. Reverse-causality chains [Pathway]
[20] Anxiety*, depression, loneliness, emotional distress, low self-esteem, sadness, and feelings of rejection due to neglect.	Learning delays, poor memory (forgetfulness), weak reasoning, reading/writing difficulties, food-obsessed, impulsive, hunger-disobedient, aggressive**, socially impaired, and apathetic.	Persistent, unsatiated hunger; voices fear of starving; frequently begs or visits neighbours/events for food; roams to find meals; eats rapidly and seeks seconds; fights over food; self-rations tiny portions; and attends school only when fed [severe food insecurity^‡^]	[Poverty, caregiver upheaval, social exclusion, capacity]^a^ [Lack of psychosocial support, school meals and caregiver involvement, absenteeism, poor academic performance]^b^ [Community support, teacher interventions]^c^	Parental [both] mental health difficulties → anxiety and depression → impaired household functioning → physical hunger [Social drift]^e^
[21] Anxiety*, sadness, social isolation, low self-esteem, low self-confidence, preoccupation with many thoughts (rumination), social withdrawal, self-doubt.	Anger outbursts**, social withdrawal, lashing out, peer rejection, defiance, difficulty engaging, attention and concentration deficits, sometimes social communication deficits, uncoordinated speech, and underdeveloped language skills.	Meal skipping; single-staple diets; wasting/stunting; weakness; frequent illness; inadequate school feeding; no packed meals; no breakfast [Severe food insecurity^‡^]	[Substance abuse, poverty]^a^ [Lack of psychosocial support, strong peer relationships, poor academic performance, school programs]^b^ [Peer support, caregiver mental stability]^c^	Parental [both] mental health difficulties → anxiety and child trauma → emotional hunger [Social drift]^d^
[22] Depressed*, anxious, hopelessness, frequent crying, lost motivation, low self-esteem, self-guilt*, trauma, appetite loss, grief, emotional distress, and social withdrawal.	Avoiding peers, peer rejection**, defiance, frequent urination, headaches, and nosebleeds.	Single-staple diet (pineapple only); skipping meals; no container/packed food, sometimes only cassava or sweet potatoes, weight loss [severe food insecurity^‡^]	[Parental mental health, domestic violence, poverty]^a^ [Lack of psychosocial support, strong peer relationships, school programs]^b^ [Peer support, caregiver involvement, school resources]^c^	Parental [paternal] mental health and domestic violence → child trauma and depression → physical hunger, emotional hunger [Social drift]^e^
[23] Depressed*, anxious, self-hatred, shame, emotional distress, social isolation, absent-mindedness, sadness, guilt, low self-esteem, and social withdrawal.	Decreased concentration, decreased attention, refusal to eat in front of others, and peer mocking**	Insufficient meals (only sweet potatoes and or cassava), skipping meals, brings no packed food, severe hunger, and bodily weakness [severe food insecurity^‡^]	[Parental mental health, adverse childhood events, poverty]^a^ [Lack of psychosocial support, school programs, peer relationships]^b^ [Peer support, caregiver involvement, school interventions]^c^	Parental [paternal] mental illness → financial instability and adverse childhood events/ trauma → physical hunger, emotional hunger, depression with anxiety, academic struggles [Social drift]^e^
[27] Emotional distress, social withdrawal, depression, disrupted appetite, low self-esteem, anxiety*, and concentration difficulties.	Social withdrawal, overeating when offered food**, rejection by peers, defiance, and poor class participation.	One daily meal; begs teachers for food; arrives unfed; drinks evening-only water; eats discarded food from neighbourhoods, physical weakness [severe food insecurity^‡^]	[Mother’s mental illness, family breakdown, trauma]^a^ [Lack of psychosocial support, school programmes, peer relationships]^b^ [Peer support, school interventions, caregiver involvement]^c^	Parental [maternal] mental illness and family breakdown → childhood trauma and emotional distress → worsening food insecurity [Social drift]^d^

→ (red arrow) indicates a direct reverse causality.

→ (blue arrow) indicates an indirect reverse causality.

‡ Inferential theme extracted through abductive reasoning.

* A most dominant internalising difficulty.

** A most dominant externalising difficulty.

a Contextual themes not in a reverse-causality sequence.

b Contextual themes lying in a reverse-causality sequence.

c Contextual themes strengthening or weakening the reverse-causality interaction.

d Direct pathway.

e Indirect pathway.

Teachers reported that mental health difficulties undermined caregiving and provisioning through reduced work capacity, disrupted routines, strained family functioning, relationship breakdowns, caregiver absences, increased child-care burdens and diversion of resources to crises. Additionally, these teachers reported that the social drift pathway exacerbated multidimensional poverty and increased the vulnerability of children to food insecurity.

#### Direct social drift

Three teachers (8.3%) reported that parental mental health difficulties and caregiver substance abuse directly disrupted household food accessibility, leading to severe food insecurity and school-level consequences in children.

A 10-year-old boy in a nuclear family faced neglect from a father who drank heavily and a mother who became mentally unstable. He grew thin, pale, and weak, often dozed off in class, lost confidence, and withdrew socially; peers mocked him because of his mother’s mental illness. He never had enough food or money for breakfast and sometimes lashed out when provoked.^**6**^ (Female teacher, 48 years).

While this boy’s experience demonstrated how parental mental health difficulties destabilised caregiving and left him both neglected and hungry, another boy’s experience (see below) demonstrated the same pathway at the intersection of trauma, stigma and disrupted appetite.

A 13-year-old boy was traumatised by his mother’s mental illness and developed similar symptoms. After his father left them to remarry, the boy wandered the village, survived on discarded leftovers, and was mocked at school, which worsened his withdrawal. When food was offered, he tended to overeat, suggesting a disrupted appetite amid emotional and physical deprivation. Concentration and class participation suffered.^**7**^ (Female teacher, 46 years).

The direct social drift pathway operated through mental health difficulties, particularly among caregivers and, in some cases, children, serving as the initial and immediate cause of food insecurity. These pathways highlight how food insecurity may arise not only from material deprivation but also from psychosocial disruptions within households.

#### Indirect/mediated social drift

Five teachers (13.6%) reported that, in the social drift pathway, parental and/or child mental health difficulties did not directly lead to food insecurity in a single step. Two teachers (5.6%) reported that mental health difficulties disrupted family income streams in three food-insecure households (8.3%) and affected caregiving practices, resulting in childhood neglect in another three food-insecure households (8.3%).

Furthermore, five teachers (13.8%) reported that mental health difficulties caused social stigma and peer mockery among children from food-insecure families. Five teachers also observed negative emotional or behavioural reactions (13.8%) among food-insecure children. Four teachers (11%) saw children disengage from learning due to caregiver mental illnesses, which had left homes without food; all of these factors increased children’s vulnerability to physical and emotional hunger, parental/caregiver neglect and social stigma.

A 13-year-old boy living with grandparents after parental neglect experienced persistent hunger and constant preoccupation with food. He fought peers, faced rejection, skipped school, and fell behind academically—becoming lonelier and more aggressive in a cycle of learning poverty.^**8**^ (Female teacher, 35 years).

Although this boy’s experience showed how the absence of a caregiver and mental distress affected daily household functioning, leaving him both physically hungry and socially excluded, another boy’s experience demonstrated the same pathway, but with domestic violence and stigma making food deprivation even worse.

Following domestic violence and separation from a father exhibiting mental instability and heavy drinking, a 13-year-old boy became guilty and hopeless, lost his appetite, and often refused neighbours’ food. Severe food insecurity meant he brought only small pieces of cassava or sweet potatoes to school in a polythene bag. Peers mocked his “insane” family; he avoided others and sometimes refused to eat. His once-strong academic performance dropped sharply.^**9**^ (Male teacher, 51 years).

Another girl’s experience demonstrated how parental mental health difficulties and bereavement triggered asset depletion, reshaping her relationship with food, self-identity and learning.

A previously high-performing 8-year-old girl experienced a decline in well-being following her father’s head injury and subsequent death. As household resources were depleted to cover medical expenses, the family’s food situation deteriorated. At school, the feeding programme shifted from biscuits/Safi to boiled sweet potatoes and cassava, which the child perceived as inferior relative to her previous circumstances. This change became a source of stigma, as she felt ashamed of eating these foods in the presence of peers. Consequently, she began to avoid eating at school, leading to increased hunger, drowsiness, and reduced concentration. Over time, she became socially withdrawn, developed feelings of self-hatred, and her academic performance declined.^**10**^ (Female teacher, 29 years).

The mediated social drift pathway, operated through caregiver-related disruption, which contributed to food insecurity via multiple interacting mechanisms, involving behavioural, social, psychological and structural processes. These included school disengagement and peer exclusion, which further limited access to resources, as well as psychological problems such as appetite disruption, self-restriction and internalised stigma that shaped children’s engagement with food. In some cases, these processes were compounded by cascading structural shocks, where caregiver illness triggered economic decline (e.g., asset depletion), which was then amplified by stigma and psychological distress, further constraining food access and educational participation.

### Bidirectional relationship: Mutual reinforcement between food insecurity and child mental health

Ten teachers (27.7%) reported cases where neither condition of food insecurity nor mental health difficulties could be clearly identified as the sole initiating factor; instead, each reinforced the other through ongoing cycles of deprivation and psychological distress (see Table 3).

**Table 3.** Feedback loops/bidirectional influences between food insecurity and child mental health: inferential and dispositional themes Table 3. long description.

Inferential themes category 1 [Scenario] Food insecurity indicators (Severity)	Inferential themes category 2 Internalising difficulties [Externalising difficulties]	Dispositional theme category 1 Other contextual factors	Dispositional theme category 2 Feedback loops/Bidirectional
[24] One meal per day, most often goes to bed hungry (Severe food insecurity^‡^)	Anxious*, social withdrawal [Aggressive**, truancy]	Substance abuse, family instability	Parental mental health difficulties (psychosis) → imitative behaviour, physical and emotional hunger ↔ emotional distress and aggression ↔ truancy (missed classes) and academic decline → school dropout
[25] Usually skips meals, often collapses due to extreme hunger (Severe food insecurity^‡^)	Sad, severely stressed*, social withdrawal, depressed mood [bullied, aggression, conduct problems**, delinquency]	Parental separation, family instability, and neglect	Childhood trauma → food refusal (through appetite loss) ↔ depressed mood, emotional hunger ↔ social isolation → academic decline
[26] Always hungry, eating pigsty leftovers, scavenging for food (Severe food insecurity^‡^)	Stressed, depressed*, socially isolated, mocked and bullied [Stealing from others, aggression]	Caregiver neglect, family instability	Physical hunger → emotional distress, withdrawal ↔ peer rejection → school absences (truancy) → academic decline
[28] Monotony of cassava/porridge, one meal a day, frequent hunger (Severe food insecurity^‡^)	Sad*, anxious, social withdrawal [begging others, stealing from others**]	Parental absences and neglect, child labour	Parental mental health difficulties → physical hunger↔ emotional distress ↔ emotional hunger, begging and social isolation → school absences (truancy) → academic decline
[29] Hungry at home, marijuana use to cope with hunger (Severe food insecurity^‡^)	Worried, social withdrawal, isolation* [marijuana use, peer mockery**]	Substance use, parental neglect, and family instability	Physical hunger↔ marijuana use (to suppress hunger) ↔ social isolation → poor grades, academic decline
[30] Begging for food (Moderate food insecurity^‡^)	Hopeless*, social withdrawal, suicidal ideation [Begging others, missed classes (truancy**)]	Parental neglect	Physical hunger → emotional distress, emotional hunger ↔ suicidal thoughts → academic decline → school withdrawal
[31] One meal a day, served less food (Severe food insecurity^‡^)	Worried*, sad, depressed, low self-esteem [Begging others**]	Parental separation, orphanhood status	Parental mental health difficulties → physical hunger, emotional hunger, emotional distress ↔ poor concentration, academic decline ↔ social withdrawal → school dropout
[32] One meal a day; does not come with food to school (Severe food insecurity^‡^)	Depressed*, low self-esteem [Wandering]	Parental loss, domestic abuse, and homelessness	Physical hunger↔ emotional trauma → social withdrawal → school dropout
[33] Underfed at home, often hungry; shamed for stealing food at school (Severe food insecurity^‡^)	Anxious*, depressed, isolated [stealing from others**]	Risky sexual behaviour, family neglect, and child labour	Sexual Abuse → emotional distress ↔ food theft, maladaptive behaviour ↔ social isolation → academic decline → school dropout
[34] Food withheld by caregiver (Moderate food insecurity^‡^)	Emotional distress*, social withdrawal [Stealing from others**, aggressive]	Family instability, discrimination	Emotional hunger → disruptive behaviour, stealing others’ food, psychological distress ↔ academic decline

→ (black arrow) indicates direct causality.

↔ (red double-headed arrow) indicates a feedback loop.

‡ Inferential themes extracted through abductive reasoning.

* The most dominant internalising difficulty.

** The most dominant externalising difficulty.

Teachers associated meal irregularity with physical hunger, which was then worsened by the stigma of eating non-preferred foods over time. Additionally, teachers reported that emotional hunger had resulted from depressed mood and behavioural problems, and later caused strained relationships, decreased classroom engagement and reduced peer support. Furthermore, teachers also reported that the lack of access to desired foods causes children to develop harmful coping strategies against food insecurity (e.g., engaging in substance and/or drug abuse, survival sex), as well as self-harm (through deliberately refusing to eat) and suicidal behaviours (e.g., deliberately drinking paraffin or swallowing overdose drugs).

A 12-year-old boy lived with a mother who developed psychosis after starting anti-retroviral medication. The family became severely food insecure; the father drank or smoked cannabis and even encouraged the boy to smoke to suppress hunger. Mocked at school, the boy grew more aggressive, missed classes, and his grades fell from high to below average.^**11**^ (Female teacher, 36 years).

While this boy’s experience demonstrated how caregiver mental health difficulties and physical hunger co-evolved in a reinforcing cycle of aggression and academic decline, another girl’s experience has demonstrated the same bidirectional spiral manifesting through trauma, survival sex and educational dropout.

A 13-year-old orphan girl living with a stepmother engaged in sex work was coerced into survival sex to obtain food. Severe hunger precipitated trauma, persistent sadness, depression, social withdrawal, sharp academic decline, and dropout—a bidirectional spiral of deprivation and psychological harm.^**12**^ (Male teacher, 40 years).

Whereas this girl’s experience demonstrated how hunger and trauma reinforced each other through survival strategies, another boy’s experience underscored how the psychological pain of being food insecure escalated into suicidal behaviour.

A 13-year-old boy suffering from moderate food insecurity, neglected by his father, begged for food at school and withdrew socially. His distress over not having desired foods escalated to a suicidal act (swallowing seven Panadol tablets), with hunger and emotional pain intensifying each other.^**13**^ (Male teacher, 34 years).

Unlike the boy’s suicidal act, another girl’s experience showed how trauma, shame and food deprivation are embedded in daily survival behaviours, trapping the children in a negative, continuous feedback loop.

A 12-year-old girl traumatised by her father’s sexual assault and her stepmother’s refusal to provide food developed anxiety, isolation, and depression. She often stole jackfruit to survive, felt shame and emotional absence, and eventually dropped out; psychological trauma and food deprivation mutually worsened each other.^**14**^ (Female teacher, 32 years).

The bidirectional pathway operated through reinforcing cycles, in which behavioural, social, structural and emotional processes jointly intensified both food insecurity and mental health difficulties. Initial disruptions, whether psychological or material, rapidly evolved into recursive dynamics in which each condition amplified the other over time. In some cases, survival strategies adopted in response to food insecurity became embedded within cycles of trauma and deprivation, reinforcing psychological distress and continued vulnerability. In others, food insecurity and psychological distress were closely intertwined at the experiential level, with emotional responses to hunger directly intensifying both mental health difficulties and vulnerability. These dynamics were further compounded in cases where trauma and food deprivation co-occurred, becoming mutually reinforcing, with psychological distress both shaping and being shaped by ongoing material deprivation.

### Educational consequences: Attendance, achievement, core skills and extracurricular engagement

Thirty-six teachers consistently observed that food insecurity and mental health difficulties among parents or caregivers were associated with significant disruptions in schooling. Of the 36 teachers, 32 (89%) reported declines in academic performance and/or achievement, while 28 (78%) observed declines in language and competency skills. Additionally, 26 (72%) reported increased school absenteeism, and 17 (47%) reported reduced participation in extracurricular and talent activities among children experiencing food insecurity and mental health difficulties.

Most importantly, the type of hunger and the severity of food insecurity were reported to have intersected while shaping child mental health difficulties and academic outcomes (see Supplementary Table S2).

Beyond the broader cycles of food insecurity and mental health difficulties, two teachers (5.6%) reported how intra-household practices, such as deliberately reducing food portions or failing to share food equally, directly undermined children’s learning. Teachers reported that these subtle yet harmful dynamics demonstrate why food insecurity is not only about scarcity, but also about how food is rationed within families, with profound consequences for academic performance, as depicted in this boy’s experience below.

An 11-year-old boy initially received ample packed food, but as his grades slipped, his mother reduced his portions to just a few sweet potatoes. He became constantly hungry, dozed during afternoon lessons, and lost concentration. Begging from classmates led to bullying and isolation, loss of friends, reduced motivation, a sharp academic decline, and worsening language skills. (Female teacher, 29 years).

### Contextually grounded explanatory model

**Figure 2.**
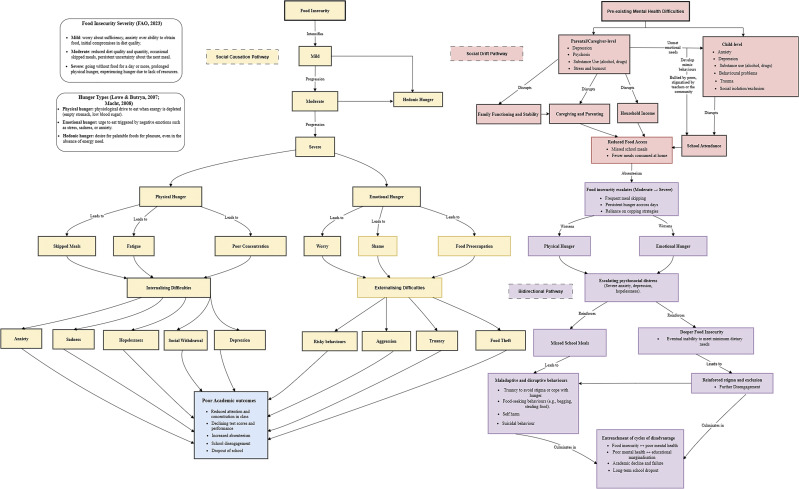
Contextually grounded explanatory model connecting social causation, social drift and bidirectional pathways. Figure 2. long description.

Figure 2 presents an explanatory model that depicts how food insecurity and child mental health difficulties interact through three interconnected pathways – *social causation*, *social drift* and *bidirectional feedback loops.* The explanatory model synthesises insights from teacher-reported child scenarios, thematic coding and realist analytical reasoning, shedding light on how these processes unfold dynamically within resource-limited, last-mile school environments.

In the *social causation pathway*, food insecurity acts as the initiating factor leading to mental health difficulties. Children experiencing *mild* to *severe food insecurity* endure both *physical hunger* (physiological deprivation due to skipped meals or inadequate diet) and *emotional hunger* (anxiety, worry, or shame related to food insufficiency). These experiences precipitate internalising problems such as *anxiety*, *sadness* and *hopelessness*, and externalising problems such as *aggression*, *truancy* and *food theft.* Over time, these psychosocial challenges compromise *attention*, *concentration* and *school engagement*, contributing to *academic decline* and, in some cases, *dropout.*

In the *social drift pathway*, pre-existing mental health difficulties – whether in children or caregivers – undermine daily functioning and access to food. Child *depression*, *trauma*, or behavioural dysregulation often leads to *school absenteeism*, *reducing access to school meals* and further deepening food insecurity. Similarly, caregiver distress, illness, or substance use disrupts *household stability*, pushing families from *moderate* to *severe food insecurity.* This downward “drift” demonstrates how psychosocial distress can exacerbate material scarcity.

The *bidirectional pathway* captures recursive feedback loops where food insecurity and mental health difficulties reinforce one another. *Physical hunger* can trigger *shame*, leading to *withdrawal* or *truancy*; absenteeism then reduces *access to school meals*, worsening both hunger and psychosocial distress. These cycles accumulate over time, entrenching disadvantage and widening educational inequality.

This explanatory model emphasises the importance of integrated interventions that simultaneously enhance food security and nutrition, provide school- and community-based mental health support and address broader structural inequalities to break the cycles of food insecurity and poor child mental health.

### Teacher strategies to support children experiencing food insecurity

Six teachers (16.7%) emphasised that compassionate and inclusive practices, such as sharing food, assigning leadership roles and involving children in school gardens, could transform the experiences of food-insecure pupils. These small yet intentional acts not only alleviated physical hunger but also restored self-worth, improved attendance and promoted psychological stability among children struggling with neglect, trauma or chronic food insecurity.

A female teacher (aged 57) remarked that “sharing the limited food available to staff with children could be beneficial,” showing how small acts of compassion supported a neglected, food-insecure boy. Another teacher (aged 55) added that giving such children responsibility, such as serving as food prefects or garden managers, improved attendance and confidence, while a third (aged 46) noted that leadership roles, such as class monitor, “could restore self-worth and psychological stability,” even for those experiencing trauma and physical/emotional hunger.

## Discussion

This study demonstrates that food insecurity and child mental health difficulties interact in cyclical, mutually reinforcing ways within resource-limited school environments. Using realist TA, the findings show that food insecurity operates not only as nutritional deprivation but also as a psychosocial and structural condition affecting children’s mental health, behaviour and learning (Frongillo et al., 2024). The explanatory model is operationalised by using initiating conditions and temporal sequencing to classify observed chains of interactions in teacher-reported child scenarios, thereby identifying distinct entry points for intervention and hypothesis generation.

Moving beyond established associations, the findings provide a contextually grounded account of how these phenomena co-occur in everyday contexts. The identification of social causation, social drift and bidirectional pathways offers a structured way of interpreting these interactions, with pathways distinguished by differences in initiating conditions and temporal sequencing and mechanisms referring to processes such as hunger, stigma, caregiving disruption and behavioural adaptations. Importantly, similar outcomes may arise across pathways but reflect distinct underlying temporal and directional processes.

The distinction between pathways and hunger typologies is not merely descriptive, but has direct implications for intervention design and hypothesis generation. While similar outcomes may emerge across pathways, the initiating conditions and temporal sequencing through which these outcomes develop suggest different entry points for action. In the social causation pathway, where food insecurity precedes psychological distress, interventions that improve reliable, stigma-free access to food, such as school feeding programmes, may have downstream benefits for mental health and educational engagement. In contrast, in the social drift pathway, where mental health difficulties undermine caregiving capacity and food access, interventions targeting caregiver or child mental health may indirectly improve nutritional outcomes. In bidirectional contexts, where food insecurity and psychological distress co-evolve, integrated approaches that simultaneously address nutritional and psychosocial needs may be required to disrupt reinforcing cycles. Similarly, distinguishing between hunger typologies may inform more precise hypotheses in longitudinal research – for example, whether early emotional hunger predicts subsequent internalising difficulties, or whether escalating physical hunger is associated with more severe behavioural problems over time. Taken together, these distinctions do not imply rigid categories but rather provide a mechanism-informed explanatory model for identifying context-specific intervention strategies and generating testable hypotheses across diverse settings.

In the social causation pathway, food insecurity generated both hunger-related stress and food-related stigma, which undermined children’s mood, behaviour and classroom engagement, patterns consistent with global evidence connecting food insecurity to anxiety, depression and behavioural problems (Whitaker et al., 2006; Weaver and Hadley, 2009; Pourmotabbed et al., 2020; Cain et al., 2022). This study advances the literature by specifying how distinct hunger typologies operate as mediating mechanisms within the social causation pathway. Emotional hunger emerged early, manifesting as worry, shame and persistent preoccupation with food (Macht, 2008). As food insecurity intensified, physical hunger became more pronounced and was associated with more severe internalising and externalising difficulties (Stevenson et al., 2023). Hedonic hunger appeared primarily at mild-to-moderate levels of food insecurity, reflecting reward-driven food desire in the absence of physiological need (Lowe and Butryn, 2007).

In the social drift pathway, preexisting parental or caregiver mental health difficulties contributed to worsening food insecurity by disrupting household functioning, income and school participation. These findings corroborate longitudinal research showing that psychological distress can erode family routines and reduce a household’s resilience to food insecurity (Kestler-Peleg et al., 2015; Weaver et al., 2015; Ridley et al., 2020; Prati, 2024). Teachers’ narratives illustrated how drift processes manifested in daily school life: depressive symptoms, trauma or behavioural dysregulation led to absenteeism, which reduced access to meals and intensified vulnerability.

A key contribution of this study is the documentation of bidirectional feedback loops in which mental health difficulties amplify one another and co-evolve over time. These negative, recurring cycles, in which hunger triggers shame, shame prompts truancy, truancy reduces access to meals and missed meals worsen emotional and physical hunger, produce compounding psychosocial disadvantages that extend to academic disengagement and dropout. This pattern aligns with broader poverty–mental health frameworks (Patel et al., 2018; Ridley et al., 2020) and provides context-specific empirical insight into how such cycles develop in a resource-limited setting. By unpacking these mechanisms within school and household contexts, the findings contribute to a more nuanced understanding of how experiences of food insecurity and mental health difficulties accumulate over time, in children based in resource-limited settings. In addition, the findings further demonstrate that food insecurity operates not only as a nutritional condition but also as a psychosocial experience shaped by hunger-related stress, food-related stigma and social interaction within school environments. In parallel, mental health difficulties, particularly among caregivers, can constrain children’s access to food by disrupting household functioning, caregiving capacity and school participation. Together, these dynamics underscore the importance of understanding food insecurity and mental health as interconnected, multidimensional processes.

Negative learning consequences were woven into these pathways, explaining why learning poverty remains pervasive. Teachers consistently associated food insecurity with decreased attention span, increased absenteeism, behavioural dysregulation and reduced participation, findings consistent with prior evidence on the association between food insecurity and academic and socio-behavioural problems (Jyoti et al., 2005; Florence et al., 2008; Shankar et al., 2017). These further heighten academic disengagement and age-for-grade heterogeneity in resource-limited school environments. This study combined hunger typologies with food insecurity levels, generating context-specific mechanisms that may be relevant to similar resource-limited settings and warrant further empirical investigation on how food insecurity and mental health difficulties together lead to educational marginalisation worldwide.

Although broader structural factors such as poverty and economic instability were reflected in teacher narratives, these were experienced by children through proximal mechanisms, including hunger, caregiving instability and school-level deprivation. These structural pressures heightened the risk of children becoming vulnerable to both food insecurity and mental health difficulties. For instance, teacher narratives showed how climate variability or seasonal changes, economic instability and caregiving problems were experienced as conditions that co-occurred with inadequate food, leading to physical hunger, food-related stigma, survival behaviours and declining academic engagement (Trudell et al., 2021; Kasujja et al., 2025). These patterns align with emerging evidence from East and West Africa documenting the centrality of stigma, rejection and mental health in the lived experiences of food insecurity (Jesson et al., 2021; Posey et al., 2024; Lang’at et al., 2025).

### Strengths and limitations

This study addresses critical gaps identified in global and Africa-specific systematic reviews, which emphasise the scarcity of school-based, child-centred research (Trudell et al., 2021; Kasujja et al., 2025). Teacher-reported child scenarios provided ongoing observations of children, enabling analysis of how food insecurity and mental health difficulties develop over time.

Methodologically, realist TA enabled the systematic linkage of lived experiences with underlying mechanisms that fed into pathways (Wiltshire and Ronkainen, 2021) within a contextually grounded explanatory model, while member checking enhanced interpretive validity (Birt et al., 2016). Rotating double-coding and documented decision rules strengthened transparency and credibility (Maxwell, 2010).

Limitations include the limited generalisability inherent in qualitative designs, the potential under-representation of internalised difficulties that are less visible to teachers, and the inability to quantify the strength of identified pathways. Although child-centred measures, such as the School-based Food Insecurity Experience Scale (formerly known as the Day Scholars Food Insecurity Experience Scale), are promising (Kasujja et al., 2023), they were not used in this qualitative study. They should be incorporated into future mixed-methods studies.

A further limitation is the reliance on teacher-reported perspectives obtained through FGDs, without direct inclusion of children or caregivers. While teachers provided valuable, sustained observations of children’s experiences within school settings, this approach may not fully capture individual-level experiences or household dynamics shaping food insecurity and mental health. Incorporating multi-informant perspectives, particularly through in-depth interviews with caregivers and children, could provide additional depth and enable triangulation of findings. Future research should therefore consider mixed-method and multi-informant designs to more comprehensively examine these processes across school and household contexts, because internalising symptoms may have been concealed in teacher-reported accounts.

### Recommendations for future research, policy and practice

The findings suggest that addressing these intertwined processes may require coordinated approaches that engage multiple domains, including school-based food provision, psychosocial support and broader social protection systems. However, given the exploratory and context-specific nature of this study, these implications should be interpreted with caution. Additionally, the distinction between pathways may also have implications for future hypothesis generation and study design. For example, longitudinal research could examine whether different initiating conditions (e.g., food insecurity versus caregiver mental health difficulties) lead to distinct developmental trajectories, or whether bidirectional processes are more strongly associated with persistent disadvantage over time.

Further research is needed to assess how these mechanisms operate across different contexts and to determine the effectiveness of integrated intervention approaches. Similarly, longitudinal or mixed-methods designs that test the causal pathways identified in this study through temporal ordering are needed to quantify how food insecurity, mental health difficulties and negative academic outcomes influence one another (Ridley et al., 2020). Because the explanatory model developed in this study is intended as a context-specific analytical tool rather than a generalisable framework, it provides a structured way to interpret complex, overlapping processes observed in this low-resource setting and may serve as a basis for further empirical investigation in similar contexts.

These findings also suggest that caregiving contexts may shape how children experience and express food insecurity. For example, children in food-insecure households with stable and supportive caregivers may appear to experience less severe psychological consequences than those exposed to caregiving disruption. However, such differences may also reflect broader contextual factors beyond food insecurity itself, including variations in caregiving quality, household stability and social support. As such, the potential moderating or mediating role of caregiver well-being cannot be inferred directly from the present data and warrants further investigation through longitudinal and comparative research designs.

Measurement innovation is urgently needed to more accurately capture the multidimensional nature of food insecurity as experienced by children. In particular, emotional hunger, which is characterised by feelings of anxiety, shame and preoccupation with food and food-related stigma, emerged in this study as a central driver of psychological distress and school disengagement, yet remains largely underrepresented in existing survey instruments. Including validated child-centred tools, such as the School-based Food Insecurity Experience Scale (Kasujja et al., 2023), and creating stigma- and hunger-sensitive measures would enhance monitoring systems. The findings suggest that these overlooked dimensions are not peripheral, but may play a key mechanistic role in connecting food insecurity to internalising and externalising difficulties. For example, experiences of stigma associated with not having food, or bringing non-preferred foods, were closely tied to peer rejection, withdrawal and reduced classroom participation. Similarly, emotional responses to hunger, including persistent worry or distress, appeared to shape behavioural coping strategies and engagement in learning.

Incorporating these constructs into measurement tools, particularly through child-centred, contextually adapted instruments, could substantially improve the sensitivity of research and monitoring systems to detect early psychosocial impacts of food insecurity. Future studies should therefore prioritise the development and validation of measures that capture emotional and social experiences of hunger, alongside traditional indicators of food access. Such advances would not only strengthen empirical research but also support the design of interventions that more effectively address both the nutritional and psychosocial dimensions of food insecurity, within global child-wellbeing frameworks, including CAP-2030 (Coalition for Children and Adolescents 2030, 2024) and the WHO – UNICEF – Lancet Commission (UNICEF et al., 2020). Therefore, these findings reinforce the need for measurement approaches that move beyond household-level indicators to capture children’s lived experiences of food insecurity within school and social environments.

Intervention studies should assess school-based models that combine feeding programmes with psychosocial support and mental health services, aiming to enhance food security and child mental health. The explanatory model developed in this study may help inform such approaches by identifying different entry points and mechanisms through which interventions could operate. For example, in contexts where food insecurity appears to precede psychological distress (social causation), strengthening access to reliable and stigma-free school meals may reduce both nutritional deprivation and associated emotional and behavioural difficulties. In contrast, where mental health difficulties contribute to worsening food insecurity (social drift), interventions that support caregiver or child mental health may indirectly improve food access and household functioning. In settings characterised by bidirectional processes, coordinated approaches that simultaneously address nutritional and psychosocial needs may be required to interrupt reinforcing cycles of deprivation and distress. Universal school feeding models, which minimise food-related stigma and can be integrated with psychosocial support, may be particularly relevant in such contexts, as discussed by Bundy et al. (2018), Jomaa et al. (2011) and Weaver and Hadley (2009), and may be particularly effective in breaking the cycle of hunger and distress. However, given the exploratory and context-specific nature of this study, these implications should be interpreted cautiously and warrant further evaluation through longitudinal and intervention-based research.

These findings also suggest that effective actions are unlikely to be achieved through single-sector interventions alone. Rather, they point to the need for a concerted, multi-level approach that combines school-based food and nutrition support, family-centred psychosocial interventions and broader social protection measures. It is the interaction between these domains, rather than any single intervention, that is likely to be most effective in addressing the interconnected nature of food insecurity and child mental health difficulties in resource-limited settings.

At the policy level, coordinated social protection systems that link cash transfers, food assistance and mental health services for parents or caregivers could mitigate drift processes that push families toward severe food insecurity and psychological distress. The explanatory model developed in this study provides a mechanism-informed lens for understanding how such policies might operate across different contexts. For instance, where food insecurity is the primary driver of mental health difficulties (social causation), policies that prioritise reliable access to food through school feeding or targeted food assistance may have downstream benefits for children’s mental health and educational engagement. In contrast, where mental health difficulties among caregivers or children contribute to worsening food insecurity (social drift), integrating mental health support into social protection systems may strengthen caregiving capacity and household stability, thereby indirectly improving food access. In contexts characterised by bidirectional processes, policies that address both nutritional and psychosocial needs in a coordinated manner may be necessary to interrupt reinforcing cycles of deprivation and distress.

Rather than prescribing a single policy solution, the model highlights the importance of cross-sector alignment, suggesting that the effectiveness of social protection interventions may depend on how well they address the underlying mechanisms connecting food insecurity and mental health in specific settings with limited resources. These implications should be interpreted cautiously and warrant further empirical investigation across diverse contexts.

## Conclusion

This study shows that food insecurity and child mental health difficulties are intertwined through social causation, social drift and bidirectional pathways, shaping children’s well-being and educational engagement in resource-limited school settings. Findings from teacher FGDs highlight how physical and emotional hunger, varying with the severity of food insecurity, contribute to internalising and externalising difficulties that hinder learning and classroom participation. These pathways are distinguished by differences in initiating conditions and temporal sequencing, providing a structured way to interpret how similar outcomes may arise through different underlying processes. In doing so, the study extends existing evidence by shedding light on how these reinforcing cycles unfold in everyday contexts.

Breaking these cycles requires integrated approaches that combine food security, nutrition and psychosocial support with broader social protection. While schools are important entry points, sustainable progress depends on coordinated, multisectoral actions that address underlying socioeconomic vulnerability and strengthen caregiving environments. The explanatory model offers a structured, mechanism-informed basis for future longitudinal and intervention research, supporting the design of integrated, multi-level strategies aligned with context-specific drivers of vulnerability. Such approaches, which recognise the interaction between nutritional, psychosocial and structural factors, are likely to yield more meaningful and sustained improvements than single-sector responses.

## Supporting information

10.1017/gmh.2026.10232.sm001 Kasujja et al. supplementary material 1 Kasujja et al. supplementary material

10.1017/gmh.2026.10232.sm002 Kasujja et al. supplementary material 2 Kasujja et al. supplementary material

## Data Availability

The focus group data generated and analysed during the current study contain sensitive and potentially identifiable information about the teacher participants. In accordance with ethical approval granted by the King’s College London Health Faculties (Blue) Research Ethics Subcommittee, these data cannot be made publicly available. Anonymised excerpts relevant to the study’s findings are included within this article, and further details may be obtained from the corresponding author upon reasonable request.

## Supplementary material

The supplementary material for this article can be found at http://doi.org/10.1017/gmh.2026.10232.

## Long descriptions

### Figure 1. Long description

At the top, three boxes are aligned horizontally. From left to right: Phase 1 Data Familiarisation describes transcript reading and annotation for understanding teacher-reported scenarios; Phase 2 Thematic Coding using Realist T A involves double coding with three theme levels using inductive, abductive, and retroductive reasoning; Phase 3 Framework Construction assigns pathways. All three phases point downward to three vertically stacked theme levels: Level 1 Experiential Themes (inductive, descriptive, data-proximal patterns), Level 2 Inferential Themes (abductive, interpretive cross-scenario patterns), and Level 3 Dispositional Themes (retroductive, causal chains, reverse-causality chains, bi-directional feedback loops). Each theme level connects rightward to a corresponding pathway: Pathway 1 Social Causation, Pathway 2 Social Drift, and Pathway 3 Bidirectional Relationships. All three pathways converge rightward into a single diamond-shaped box labeled Integrated Conceptual Framework. At the bottom, a horizontal bar labeled Cross-Cutting Quality Assurance at each phase lists Reflexivity, Nine Consensus Meetings, Member Checking with all 36 teachers, Inter-coder reliability (e.g., 80 percent agreement), and Decision-Rules Logs.

Navigate back to Figure 1..

### Table 1. Long description

Starting from the top row, the table has five columns: food insecurity indicators (severity), internalising difficulties, externalising difficulties, other contextual variables, and causal chains. Each row details a scenario of food insecurity, such as ‘goes hungry to bed, comes to school hungry, steals food, no food from home,’ and links it to mental health outcomes like anxiety, hopelessness, arguments, fighting, and disruptive behaviour. Contextual variables include parental illness, household instability, social exclusion, peer avoidance, and teacher punishment. Causal chains describe direct and indirect pathways, for example, ‘physical hunger leads to anxiety, irritability, arguments, food theft, social withdrawal, academic decline.’ Subsequent rows present variations, such as skipping meals, eating only supper, bringing only boiled cassava, or eating monotonous diets, each associated with specific internalising (sadness, mutism, suicidal thoughts, low self-esteem, emotional withdrawal) and externalising difficulties (defiance, truancy, begging, food theft, aggression). Contextual variables expand to include parental neglect, family disruption, bullying, peer mockery, teacher response, and child labour. Causal chains trace how hunger and emotional deprivation result in maladaptive behaviours, academic decline, social exclusion, and school dropout. Footnotes clarify dominant difficulties, causal sequences, and pathway types, with red arrows indicating direct causality and blue arrows indicating indirect causality. The table systematically maps the interplay between food insecurity severity, psychological symptoms, behavioural responses, environmental factors, and causal mechanisms across 19 distinct scenarios.

Navigate back to Table 1..

### Table 2. Long description

Starting from the top row and moving downward, each row presents a scenario with internalising difficulties such as anxiety, depression, loneliness, sadness, and low self-esteem, followed by externalising difficulties including learning delays, poor memory, impulsivity, aggression, and social impairment. The third column details food insecurity indicators like persistent hunger, meal skipping, rapid eating, single-staple diets, and severe food insecurity, often marked by physical weakness and frequent illness. The fourth column lists dispositional themes such as poverty, caregiver upheaval, social exclusion, lack of psychosocial support, poor academic performance, and community or peer support. The fifth column describes reverse-causality chains, showing pathways from parental mental health difficulties through anxiety, depression, trauma, and impaired household functioning to physical and emotional hunger, with distinctions between direct (red arrow) and indirect (blue arrow) causality. Footnotes clarify dominant difficulties, abductive reasoning, and the role of contextual themes in causality sequences.

Navigate back to Table 2..

### Table 3. Long description

Starting from the top row, each scenario of food insecurity is listed in the first column, ranging from ‘one meal per day, most often goes to bed hungry’ to ‘food withheld by caregiver.’ Severity is noted as severe or moderate. The second column details internalising difficulties such as anxious, sad, depressed, social withdrawal, and externalising difficulties like aggression, truancy, stealing, and begging. The third column identifies contextual factors including substance abuse, family instability, parental separation, neglect, child labour, homelessness, risky sexual behaviour, and discrimination. The fourth column describes feedback loops and causal pathways using arrows: black arrows indicate direct causality, red double-headed arrows indicate bidirectional influences. For example, parental mental health difficulties lead to imitative behaviour, physical and emotional hunger, which feed back into emotional distress and aggression, resulting in truancy and academic decline, ultimately causing school dropout. Other pathways include childhood trauma leading to food refusal and depressed mood, physical hunger causing emotional distress and peer rejection, and emotional hunger resulting in disruptive behaviour and academic decline. Each row presents a distinct scenario, mental health outcome, contextual factor, and feedback loop, illustrating complex interconnections between food insecurity and child mental health.

Navigate back to Table 3..

### Figure 2. Long description

At the top center, food insecurity branches into mild, moderate, and severe, with a social causation pathway leading to hedonic hunger. To the right, pre-existing mental health difficulties connect via the social drift pathway to personal or caregiver-level factors, family functioning, parenting, and household income, which lead to reduced food access and school attendance. From food insecurity, arrows descend to physical and emotional hunger, each leading to outcomes: physical hunger links to skipped meals, fatigue, poor concentration, and internalizing difficulties such as anxiety, sadness, hopelessness, social withdrawal, and depression. Emotional hunger leads to worry, shame, externalizing difficulties, risky behavior, aggression, truancy, and food theft. Both hunger types and psychosocial difficulties converge on poor academic outcomes, including reduced attainment, attendance issues, disengagement, and dropout. On the right, a feedback loop shows food insecurity escalation, missed meals, and psychosocial distress leading to deeper food insecurity, maladaptive behaviors, reinforced stigma, exclusion, and entrenchment of disadvantage, with long-term mental health and educational impacts.

Navigate back to Figure 2..
