# Supplementary material for: Food insecurity and child mental health in Masaka District, Uganda: Qualitative study using a realist thematic analysis
Source: Glob Ment Health (Camb). 2026 May 22;13:e123. doi: 10.1017/gmh.2026.10232 (PMC13279967; doi:10.1017/gmh.2026.10232)
Supplement: Kasujja et al. supplementary material 1 — Kasujja et al. supplementary material [file S2054425126102325sup001.docx]

**Supplementary Table S1. Initiating conditions and temporal sequence in each quoted child scenario reported by the teachers**

| **Superscript** | **Initiating Condition** | **Temporal Sequence** |
| --- | --- | --- |
| 1 | **Initiating condition:** Severe household food insecurity resulting in chronic daily hunger. | **Temporal sequence:** Persistent hunger → anxiety and irritability → conflict with peers and food-seeking behaviours (e.g., stealing) → teacher punishment and peer avoidance → social isolation and reduced self-confidence → increased absenteeism → academic decline and school withdrawal. |
| 2 | **Initiating condition:** Severe household food insecurity leading to repeated meal skipping (breakfast and lunch). | **Temporal sequence:** Chronic hunger and unmet food needs → food-seeking behaviours (stealing) and conflict → escalating anxiety when food is unavailable → extreme psychological distress (self-harm attempt) → engagement in labour to obtain food → reduced school attendance → academic decline, grade repetition, and eventual dropout. |
| 3 | **Initiating condition:** Severe household food insecurity and unmet food needs. | **Temporal sequence:** Food insecurity → adoption of survival strategies (stealing, affiliating with high-risk peers, engaging in risky sexual behaviours) → exposure to adverse outcomes (pregnancy) → social consequences (shame and isolation) → psychological distress and reduced academic engagement → school dropout. |
| 4 | **Initiating condition:** Chronic food insecurity resulting in daily hunger and absence of packed food. | **Temporal sequence:** Persistent hunger → heightened anxiety and fear → food-seeking behaviour (theft) → social and institutional responses (bullying, peer rejection, teacher punishment) → social isolation and reduced well-being → impaired concentration and academic engagement. |
| 5 | **Initiating condition:** Climate-related disruption to household food production (poor harvest). | **Temporal sequence:** Climate variability → reduced food production → household food insecurity → hunger and anxiety → school disengagement and academic decline. |
| 6 | **Initiating condition:** Caregiver mental health difficulties and dysfunction (father’s heavy alcohol use and mother’s mental instability). | **Temporal sequence:** Caregiver psychological instability and substance use → disruption in caregiving and neglect → reduced household food provision (lack of food and breakfast) → physical deterioration (thinness, fatigue) → psychological and behavioural difficulties (withdrawal, low confidence, irritability) → peer stigma and social marginalisation. |
| 7 | **Initiating condition:** Caregiver mental illness (mother) and resulting child psychological trauma. | **Temporal sequence:** Maternal mental illness → child trauma and emergence of similar psychological symptoms → family disruption (father’s departure) → breakdown in caregiving and supervision → severe food insecurity (reliance on discarded leftovers) → social stigma (mockery at school) → worsening withdrawal and disrupted eating patterns (overeating when food is available) → impaired concentration and classroom participation. |
| 8 | **Initiating condition:** Caregiver-related disruption and neglect associated with underlying psychosocial instability (parental neglect leading to a non-parental care arrangement). | **Temporal sequence:** Caregiver disruption/neglect → inadequate and unstable food provision (persistent hunger) → behavioural responses (preoccupation with food, fighting) → social and institutional mediators (peer rejection, school disengagement/absenteeism) → reduced access to school-based resources (including meals) → academic decline and reinforcement of vulnerability. |
| 9 | **Initiating condition:** Caregiver mental health difficulties and family disruption (father’s mental instability, substance use, and domestic violence leading to separation). | **Temporal sequence:** Caregiver instability and family breakdown → child psychological distress (guilt, hopelessness) → behavioural and emotional mediators (loss of appetite, refusal of available food) → worsening food insecurity (limited food intake despite availability) → social stigma (peer mockery) and withdrawal → reduced engagement and academic decline. |
| 10 | **Initiating condition:** Caregiver mental health deterioration following father’s head injury, leading to family disruption and bereavement. | **Temporal sequence:** Caregiver mental illness and medical crisis → economic mediator (asset depletion through sale of household possessions) → reduced household resources and change in food environment (lower-quality school food) → social and psychological mediators (peer mockery, shame, self-hatred, avoidance of eating) → reduced food intake and classroom functioning (hunger, drowsiness, disengagement) → academic decline (impaired concentration and language skills). |
| 11 | **Initiating condition:** Caregiver mental health deterioration (maternal psychosis), followed by emerging household food insecurity. | **Temporal sequence:** Caregiver mental illness → household instability and food insecurity → child exposure to hunger and maladaptive coping (e.g., substance use encouraged to suppress hunger) → behavioural responses (aggression, school disengagement) → social consequences (peer mockery, missed classes) → reduced access to school-based resources and support → worsening food insecurity and psychological distress (recursive cycle). |
| 12 | **Initiating condition:** Severe food insecurity and caregiver-related vulnerability (coercive environment linked to stepmother’s circumstances). | **Temporal sequence:** Severe food insecurity (hunger) → engagement in survival strategies (coerced sexual activity to obtain food) → psychological trauma and distress (sadness, depression, withdrawal) → social and educational consequences (isolation, academic decline) → increased vulnerability and dependence on survival strategies → sustained food insecurity and psychological harm (recursive cycle) |
| 13 | **Initiating condition:** Co-occurring food insecurity and caregiver neglect, with no single dominant starting point. | **Temporal sequence:** Food insecurity (moderate hunger) → psychological distress (shame, frustration over unmet food needs) → behavioural and social responses (begging, withdrawal) → increased social isolation and emotional pain → escalation to extreme distress (suicidal behaviour) → further psychological vulnerability and reduced engagement → sustained interaction between hunger and distress (recursive cycle). |
| 14 | **Initiating condition:** Co-occurring caregiver-related trauma (sexual assault) and food deprivation, with no single dominant starting point. | **Temporal sequence:** Caregiver-inflicted trauma and food deprivation → psychological distress (anxiety, depression, isolation) → survival behaviour (food theft) → social and emotional consequences (shame, withdrawal, emotional numbing) → reduced support and increased vulnerability → continued food deprivation and deepening psychological distress (recursive cycle) → educational disengagement and dropout. |
