# Supplementary material for: Food insecurity and child mental health in Masaka District, Uganda: Qualitative study using a realist thematic analysis
Source: Glob Ment Health (Camb). 2026 May 22;13:e123. doi: 10.1017/gmh.2026.10232 (PMC13279967; doi:10.1017/gmh.2026.10232)
Supplement: Kasujja et al. supplementary material 2 — Kasujja et al. supplementary material [file S2054425126102325sup002.docx]

| **Severity of food insecurity** | **Hunger type** | | | **Mental health difficulties**  (Teacher-reported child observations; experiential themes) | **Academic outcomes**  (Teacher-reported child observations; experiential themes) |
| --- | --- | --- | --- | --- | --- |
|  | **Physical hunger** (Teacher-reported child experiences; experiential themes) | **Emotional hunger** (Teacher-reported child experiences; experiential themes) | **Hedonic hunger** (Teacher-reported child experiences; experiential themes) |  |  |
| **Mild** | Occasional skipping of meals; slight fatigue or distraction. | Worrying about food availability; mild anxiety about the next meal. | Craving for snacks despite meals; sometimes desire for snacks or sweets despite adequate meals. | Emerging internalising difficulties, particularly mild anxiety, worry, and occasional externalising difficulties, especially irritability. | Slight distraction; reduced participation but maintains performance. |
| **Moderate** | Regularly reduced meal quantity; frequently skipping meals; tiredness and poor concentration. | Preoccupation with food; distress-driven requests for food from peers/teachers. | Increased craving for palatable foods to compensate for reduced diet quality (and consumption of non-preferred meals). | Apparent internalising difficulties, such as sadness, hopelessness, and shame, and externalising difficulties, including aggression, truancy, and food theft. | Decline in attention, slower learning, lower test scores, and increased absenteeism. |
| **Severe** | Going without food for a day or more; overt signs of hunger (stomach pain, faintness, extreme fatigue). | Persistent sadness, shame, withdrawal; behavioural coping (food theft, aggression, truancy). | Rarely observed; physiological deprivation dominated over pleasure-driven eating. | Severe internalising difficulties, particularly hopelessness, depression, and social isolation, and externalising difficulties, particularly truancy, aggression, and risky behaviours. | Academic disengagement, school dropout, loss of future educational opportunities. |

**Supplementary Table S2. Integrated matrix of severity of food insecurity, hunger type, mental health difficulties, and academic outcomes: Experiential themes**
