# Supplementary material for: Food insecurity and child mental health in Masaka District, Uganda: Qualitative study using a realist thematic analysis
Source: Glob Ment Health (Camb). 2026 May 22;13:e123. doi: 10.1017/gmh.2026.10232 (PMC13279967; doi:10.1017/gmh.2026.10232)
Supplement: Peer review history

# Reviewer report: Review: Food insecurity and child mental health in Masaka District, Uganda: Qualitative study using a realist thematic analysis — R0/PR2

**DOI:** 10.1017/gmh.2026.10232.pr2

The study addresses a timely and relevant topic. The manuscript is generally well structured and coherent. However, the contribution in terms of conceptual or empirical novelty appears limited, as the association between food insecurity and children’s mental health difficulties is already well established.

Methodologically, the reliance on 12 Focus Group Discussions (FGDs) may have constrained the depth of individual-level insights. A more balanced qualitative design – such as conducting a smaller number of FGDs (e.g., 4 FGDs from four different schools), complemented by in-depth interviews, could have mitigated some of the limitations of FGDs while capitalising on the strength of individual narratives. In addition, incorporating interviews with parents or caregivers would have enabled a more holistic understanding of household dynamics, caregiving practices, and contextual factors shaping children’s experience of food insecurity and mental health.

Furthermore, the analysis could be strengthened through a comparative perspective. For instance, comparing children from food insecure, poverty stricken households with parents who are mentally and physically stable to those included in the current study could offer a more nuanced account of how parental wellbeing mediates or moderates the relationship between food insecurity and children’s mental health difficulties. Such an approach would enhance the analytical depth of the study and contribute more meaningfully to the existing body of knowledge.

# Reviewer report: Review: Food insecurity and child mental health in Masaka District, Uganda: Qualitative study using a realist thematic analysis — R0/PR3

**DOI:** 10.1017/gmh.2026.10232.pr3

**Authors:** Thalia Sparling

Overall, I find this research intersection/topic very important as the intersection of food, nutrition and mental health not only creates cascading and compounding risk, but could also present important opportunities for synergy if we understood the dynamics better. Finding high-quality research focused directly on this topic is rare, so it’s great to see. Furthermore, well-conducted qualitative research is particularly welcome on this topic, as most quantitative primary research is not only not designed to address causal and mechanistic relationships between food security and mental health, but even if it were, there are limitations to understanding the ‘why’ and ‘how’ in any quant approach.

Below are some specific points, but the biggest concern is that the examples of the different typologies and pathways don’t seem distinct from one another. Is there a way to highlight how they are different, in ways that correspond to the different groups you have delineated? If so, that would be helpful. Overall, it doesn’t seem clear that the different typologies and pathways would lead to different intervention or actions (or even different hypothesis generation in longitudinal studies, for that matter). If that is the case (or not), maybe say a little bit about that in the discussion?

Abstract:

- Largely clear and well-described.

- Immediately I wonder about the use of ‘framework’ within this very niche population and exploratory nature of the work (although interesting). I would be cautious implying that this a universal framework, or think that intervention design changes in the broadest sense can be justified. Your findings would need to be triangulated with others to conclude that, especially in experimental testing. Maybe revise to just say these context-specific pathways could be further explored in this and similar settings with potential for improved intervention design...or something like that.

- Furthermore, the proposal for integrated interventions, such as including school-based nutrition, are a big leap from the findings highlighted in the abstract – and possibly premature based solely on this research – please try to soften the language here. After reading the whole paper, this is clearer, but the abstract should be stand-alone with everything logically linked and justified (which is challenging given tight word count).

- L38: you say ‘settings’ but it was really one setting, no?

Impact statement:

- Is 9 months ‘long term’?

- ’36 child scenarios’ is not particularly clear at this point in the paper…just say interviews with 36 teachers?

- Maybe say, ‘the conceptual framework highlighted in this context’ or something similar to qualify this as a setting/pop specific finding that may (or may not) play out in other contexts, but would need to be studied.

- The recommendations around school-based interventions is probably/obviously because this study was with teachers, in schools, but the findings seem broader than that. Maybe you could rephrase slightly to something like ‘the findings suggest that effective action in this setting would require a concert of approaches, namely improved school-based food and nutrition support, family-centred intervention and social protection…’ or something you agree with but emphasize that it’s the interplay between these realms that will be the difference?

Introduction:

- L36 – you mention cross-sectional limitations, but also add the lack of qualitative work in this space here? Set up your rationale even more?

Methods:

- This seemed like a thorough approach, especially the multiple notetaking (5!), multiple coding layers, mixed inferential approaches, and member checking. I particularly liked the elicitation of examples and stories from the teachers – instead of trying to ‘qualitatively quantify’ as sometimes happens. The framework construction made more sense after reading the methods.

Results:

- Small comment, but a couple of places authors say something like ‘About 19.4%…’ there’s nothing approximate about that;). In some ways I found the percentages less straightforward than simply stating the n, but ultimately this is your choice – one option could be to simply swap the percentages into the brackets.

- It’s not always clear how the ‘causal pathways’ are different from one another. The descriptions seem very similar, and even the progression of events in the examples given. In principle, some scenarios may work directly, whereas others work indirectly, but then what is described is often not that different, even in terms of pathways and ‘stepping stones’ to outcomes. Is it possible to highlight the differences between these more? For instance, how is the second example in 3.3.2 different from the examples in 3.3.1? Similarly, it is not apparent the differences between 3.4.1 and 3.4.2…these seem materially the same in terms of ‘directness’, although the last example of selling assets to cover hospital bills is clearly different. As an aside, the link to the school’s switch to cassava and sweet potatoes from biscuits (presumably she ate the former but refused the latter), is unclear. One last example…there are two stories about young boys and self-harm in two different sections -- it isn’t clear how these are examples of different relationships, at least to me.

Discussion:

- Without the examples, the differences between typologies and pathways make more sense, but there is clear overlap – which makes inferring the implications of these differences difficult. Not sure what to suggest, but maybe clarifying how these are different throughout could help?

- There is a mention of climate variability…I didn’t see anything about this in the examples or results. If this is evidenced, it would be good to highlight.

- The last para before 4.1 states the implications of the findings very clearly and as a singular phenomenon with a common solution – this resonates the most so far – see other comments on this.

- At the beginning of 4.1, there are a few more reviews and evidence and gap maps on this intersection that might be worth looking at to support your points as well, including in section 4.2.

- This is a very good point, and especially in LMICs: “Measurement innovation is urgently needed. Emotional hunger and food-related stigma are underrepresented in current surveys, yet have emerged as powerful drivers of mental health difficulties and academic disengagement.”

- There isn’t a lot of discussion about the framework, but that seems a big point in the abstract. Even in the results, it’s not entirely clear how this would be operationalised (see the overall point above…). Maybe you can harmonise this across abstract, results and discussion.

# Editor report: Recommendation: Food insecurity and child mental health in Masaka District, Uganda: Qualitative study using a realist thematic analysis — R0/PR4

**DOI:** 10.1017/gmh.2026.10232.pr4

**Affiliations:** Clinical Psychology, National Institute of Mental Health and Neuro Sciences Department of Clinical Psychology, India

Dear Authors,

I am happy to share that your manuscript has received independent, blinded reviews, which have suggested minor revisions. Please see the comments and suggestions and consider submitting the revised manuscript within the timeline.

Best wishes,

Thomas

# Editor report: Decision: Food insecurity and child mental health in Masaka District, Uganda: Qualitative study using a realist thematic analysis — R0/PR5

**DOI:** 10.1017/gmh.2026.10232.pr5

**Affiliations:** London School of Hygiene & Tropical Medicine, United Kingdom of Great Britain and Northern Ireland

No accompanying comment.

# Editor report: Recommendation: Food insecurity and child mental health in Masaka District, Uganda: Qualitative study using a realist thematic analysis — R1/PR7

**DOI:** 10.1017/gmh.2026.10232.pr7

**Affiliations:** Clinical Psychology, National Institute of Mental Health and Neuro Sciences Department of Clinical Psychology, India

Dear Authors,

I note that you have adequately addressed the observations and comments made by independent reviewers. I am happy to inform you that your manuscript is accepted, and you will receive separate communication from the Journal about further processes. I hope you had a pleasant experience with the review process and consider this journal for publishing your future work.

Best wishes,

Thomas

# Editor report: Decision: Food insecurity and child mental health in Masaka District, Uganda: Qualitative study using a realist thematic analysis — R1/PR8

**DOI:** 10.1017/gmh.2026.10232.pr8

**Affiliations:** London School of Hygiene & Tropical Medicine, United Kingdom of Great Britain and Northern Ireland

No accompanying comment.
